# Proteomic comparisons of opaque and transparent variants of *Streptococcus pneumoniae* by two dimensional-differential gel electrophoresis

**DOI:** 10.1038/s41598-017-02465-x

**Published:** 2017-05-26

**Authors:** Melissa H. Chai, Florian Weiland, Richard M. Harvey, Peter Hoffmann, Abiodun D. Ogunniyi, James C. Paton

**Affiliations:** 1 Research Centre for Infectious Diseases, School of Biological Sciences, The University of Adelaide, Adelaide, South Australia 5005 Australia; 2 Adelaide Proteomics Centre, School of Biological Sciences, The University of Adelaide, Adelaide, South Australia 5005 Australia; 3 Institute for Photonics and Advanced Sensing (IPAS), The University of Adelaide, Adelaide, South Australia 5005 Australia; 4 Medical Research Council Protein Phosphorylation and Ubiquitylation Unit, School of Life Sciences, University of Dundee, Dundee, DD1 5EH United Kingdom; 5 Australian Centre for Antimicrobial Resistance Ecology, School of Animal and Veterinary Sciences, The University of Adelaide, Roseworthy, South Australia 5371 Australia

## Abstract

*Streptococcus pneumoniae* (the pneumococcus) is a human pathogen, accounting for massive global morbidity and mortality. Although asymptomatic colonization of the nasopharynx almost invariably precedes disease, the critical determinants enabling pneumococcal progression from this niche to cause invasive disease are poorly understood. One mechanism proposed to be central to this transition involves opacity phase variation, whereby pneumococci harvested from the nasopharynx are typically transparent, while those simultaneously harvested from the blood are opaque. Here, we used two dimensional-differential gel electrophoresis (2D-DIGE) to compare protein expression profiles of transparent and opaque variants of 3 pneumococcal strains, D39 (serotype 2), WCH43 (serotype 4) and WCH16 (serotype 6A) *in vitro*. One spot comprising a mixture of capsular polysaccharide biosynthesis protein and other proteins was significantly up-regulated in the opaque phenotype in all 3 strains; other proteins were differentially regulated in a strain-specific manner. We conclude that pneumococcal phase variation is a complex and multifactorial process leading to strain-specific pathogenicity.

## Introduction

*Streptococcus pneumoniae* (the pneumococcus) is a formidable human pathogen, responsible for massive global morbidity and mortality. It causes a broad spectrum of diseases including pneumonia, meningitis, bacteraemia and otitis media, and accounts for more deaths worldwide than any other single pathogen^1^. In developing countries, up to 1 million children under 5 years of age die each year from pneumonia, of which *S*. *pneumoniae* is the single commonest cause, accounting for 20% of all deaths in this age group^1^. Asymptomatic nasopharyngeal carriage is an essential first step in the pathogenesis of disease^2^, and pneumococcal strains differ markedly in their capacity to invade other tissues from this niche. While certain serotypes and/or clonal groups are more often isolated from the nasopharynx, and others more often isolated from sterile sites (such as blood), the underlying genomic/phenomic differences responsible for these variations between strains are yet to be fully elucidated.

Studies of the phenomenon of colony opacity phase variation in *S*. *pneumoniae* are providing clues regarding genes that might be central to the transition from carriage to invasive disease. When *S*. *pneumoniae* colonies growing on agar plates are observed under oblique transmitted light, two distinct morphologies, described as “opaque” (O) and “transparent” (T), are observed^3^. When opaque colonies are subcultured, a small proportion of them spontaneously change to the transparent form, and vice versa^3, 4^. Interestingly, the T forms have an enhanced capacity to colonize the nasopharynx relative to O variants of the same strain, which correlates with increased *in vitro* adherence to epithelial cells, while the opaque form is associated with massively increased virulence in animal models of systemic disease^4, 5^.

The relevance of phase variation to the pathogenesis of human disease is supported by the finding that when apparently identical *S*. *pneumoniae* strains are isolated simultaneously from the nasopharynx and blood of patients with invasive disease, those from the former niche are largely in the transparent phase, whilst those from the latter are almost all opaque^3^. Recently, we reported a random six-phase genetic rearrangement in a type I restriction-modification (RM) system (SpnD39III) with distinct methylation patterns resulting in differential gene expression profiles^6^. The variants also displayed distinct phenotypic changes, including opacity phase variation differences, which have a major impact on pneumococcal virulence in mice. Variation in the levels of expression of pneumococcal capsular polysaccharide (CPS) and certain surface proteins between the two forms has also been reported^7^. However, colony opacity variation still occurs in unencapsulated mutants, suggesting that the varying amount of capsule was not entirely responsible for the colony opacity phenotype^3^. Interestingly, T variants exhibit a higher teichoic acid to capsule ratio^4^, an observation that could be of relevance given that teichoic acid is important for the anchorage of choline-binding proteins to the pneumococcal surface, thereby enhancing colonization of the nasopharynx^8, 9^. Phosphorylcholine present on the teichoic and lipoteichoic acid residues of the cell wall also interact with the platelet-activating factor receptor on respiratory epithelium, facilitating adherence to the nasopharynx^8, 10^.

We reasoned that the inconsistencies in the literature with regard to distinct expression patterns of proteins between pneumococcal colony opacity variants^3, 7, 11^ could have arisen because the pneumococcal strains and analytical techniques in each of those studies were different. In order to gain further insight into the phenotypic differences that underpin colony opacity variation in *S*. *pneumoniae*, we used 2-dimensional differential gel electrophoresis (2D-DIGE) to carry out a comprehensive comparison of protein expression profiles of T and O variants of 3 well-characterized pneumococcal strains: D39 (serotype 2), WCH43 (serotype 4) and WCH16 (serotype 6A). These strains display distinct pathogenicity profiles: D39 causes severe pneumonia and high-grade bacteremia, WCH43 demonstrates the “classical” disease progression from the nasopharynx to the lungs and dissemination to blood and then to the brain, while WCH16 seems to progress directly to the brain with minimal lung and blood involvement^12–14^. These distinct characteristics make these strains ideal for our study. We hypothesize that our analyses will identify proteins common to T and O variants of the 3 strains, while also accentuating their differences.

## Results

### Identification of opaque and transparent variants

**Figure 1:**
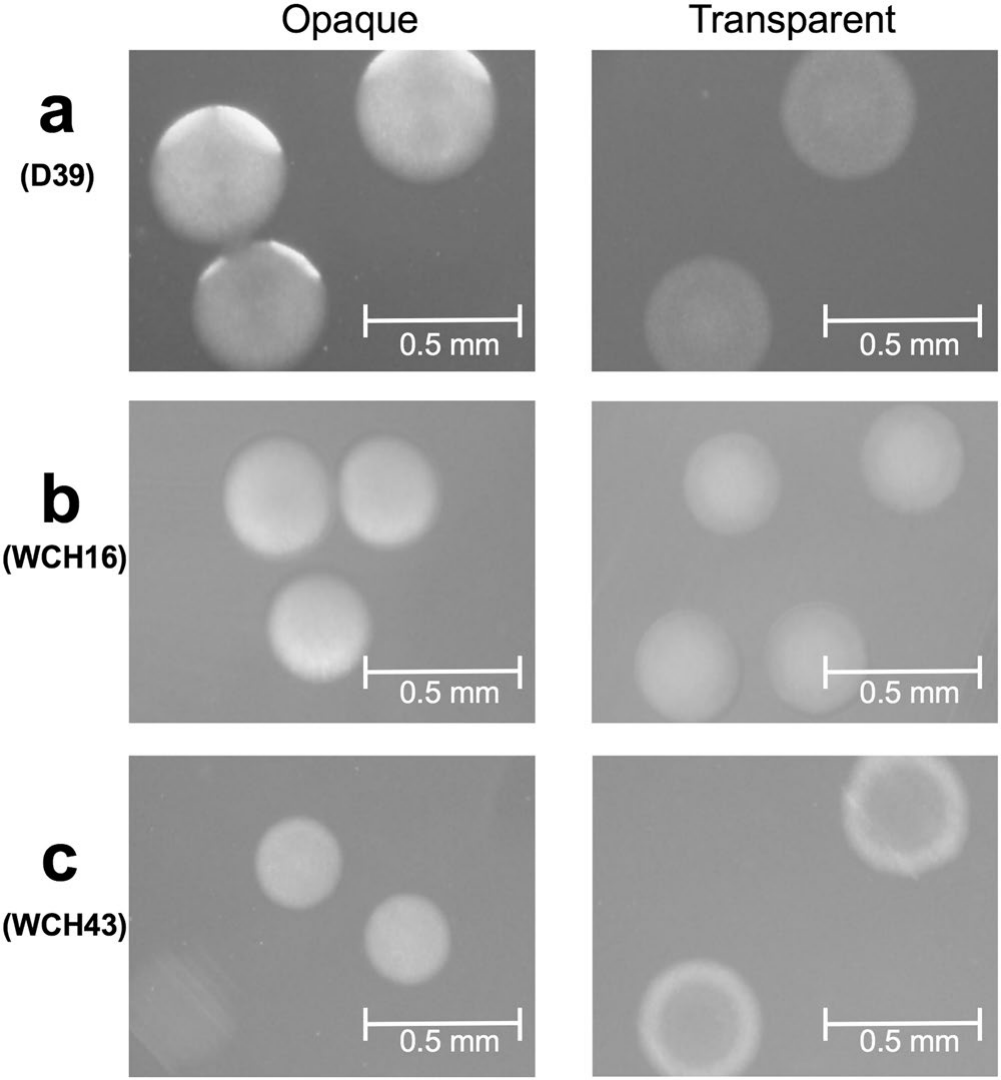
Colony morphology of various *S*. *pneumoniae* strains and their variants grown on clear media (THY) supplemented with catalase. Images were captured using Nikon SMZ1000 on a dissecting microscope and viewed through cellSens software. (**a**) D39 opaque; (**b**) D39 transparent; (**c**) WCH16 opaque; (**d**) WCH16 transparent; (**e**) WCH43 opaque; (**f**) WCH43 transparent.

Pure O and T working stocks of D39, WCH16 and WCH43 were selected on THY-catalase plates based on their appearance under oblique, transmitted light as described in Methods. We observed that the O colonies are typically of convex elevation, compared with those of T form, which have umbonate elevation, exhibiting a doughnut-like appearance (Fig. 1). We also found that the strains have markedly different incubation times by which the colony opacity could easily be observed: 18 hours for D39, 24 hours for WCH16, and 36 hours for WCH43.

### Analysis of differential protein expression profiles

To detect differentially expressed proteins between the O and T phenotype of the 3 strains, three independent DIGE experiments were performed, as described in Methods.

#### D39

**Figure 2:**
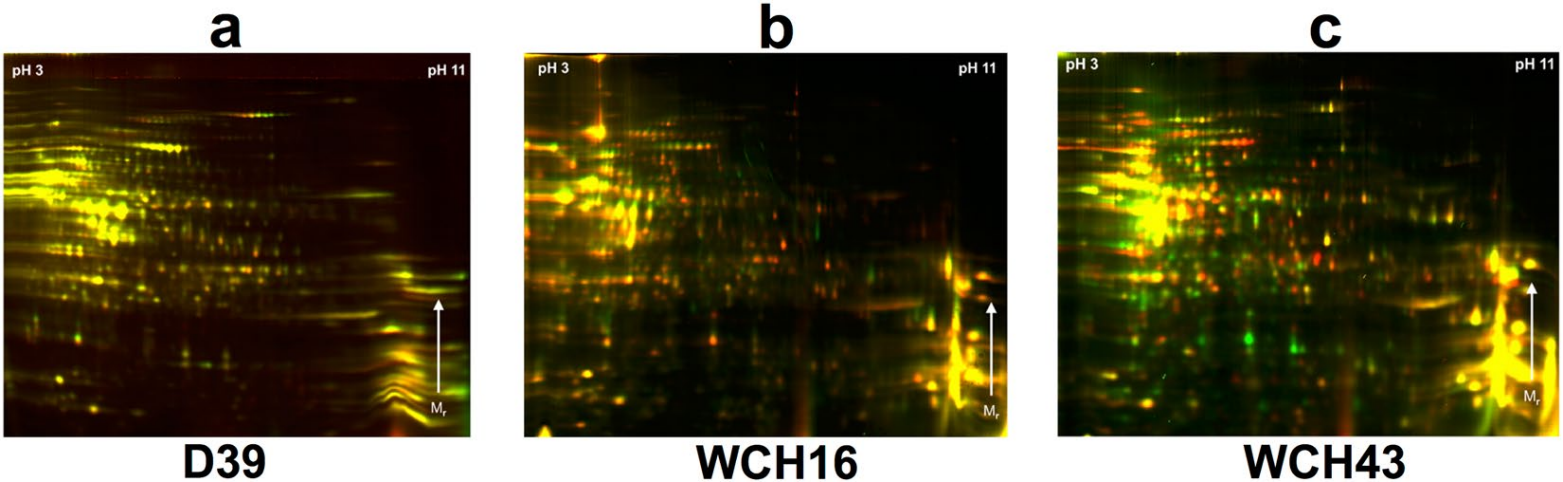
2D-DIGE analysis of protein lysates of a mixture of opaque and transparent variants of *S*. *pneumoniae* D39 (**a**), WCH16 (**b**) and WCH43 (**c**), highlighting differentially-regulated proteins between the variants. Images show false-color overlay of Cy2, Cy3 and Cy5 channels. Proteins with different signal intensities are shown either in green (Cy3) or red (Cy5) color, where yellow color indicate a similar amount of protein on the representative gels.

The gel image data from the comparison of the T (n = 4) and O variants (n = 4) of D39 by DIGE was processed by DeCyder. Spot patterns on the individual gel images of D39 were matched against a single master gel to ensure that all spots across the different gel images had the same reference number. A total of 982 protein spots were detected on the master gel, 602 of these spots were present in 75% of the spot maps (9 out of 12). Univariate statistical testing detected 65 differentially expressed spots with a *p*-value < 0.05. Of the 65 proteins, 37 protein spots were detected to be up-regulated in the O variant, while 28 protein spots were up-regulated in the T variant. The average fold-changes were between 10.9 (up-regulated in the O variant) and 15.8 (up-regulated in the T variant) (Fig. 2a; Supplementary Fig. S1). At a significance level α = 0.05, the q-value extended false discovery rate (FDR) was calculated to be 13%. *Post*-*hoc* power calculation resulted in the ability to detect a fold-change of 2.5 and above with at least 80% probability.

#### WCH16

Detection of differentially expressed proteins between the O (n = 4) and T variants (n = 4) of WCH16 was performed as described for D39 above. In total, 1168 protein spots were detected on the master gel, and 674 of these could be detected across 75% of the spot maps. Statistical testing detected 73 proteins as differentially expressed with a significance level α = 0.05. Of these, 46 protein spots were up-regulated in the O variant and 27 protein spots were up-regulated in the T variant. Average fold-changes were calculated to be between 3.7 (up-regulated in the O variant) and 4.9 (up-regulated in the T variant) (Fig. 2b; Supplementary Fig. S2). The corresponding q-value extended FDR was calculated to be 66%. *Post*-*hoc* power calculation resulted in the ability to detect fold-changes of 2.1 and above with at least 80% probability.

#### WCH43

The O (n = 4) and T (n = 4) variants of WCH43 were also compared for differential protein expression as described above. On the master gel, 1285 protein spots could be detected of which 879 were present in 75% of all spot maps. The statistical testing resulted in 183 differentially expressed proteins, with a cut-off value of α = 0.05. As with the other *S*. *pneumoniae* strains investigated in this study, there were more up-regulated protein spots in the O variant (n = 118) than in the T variant (n = 65). The q-value extended FDR for this significance level of α = 0.05 was 11%. *Post*-*hoc* power was calculated and resulted in the ability to detect a fold-change of 2.5 and above with at least 80% probability. The average fold-change of the differentially expressed proteins ranged between 7.5 (up-regulated in the O variant) and 18.2 (up-regulated in the T variant) (Fig. 2c; Supplementary Fig. S3).

### Pneumococcal strains exhibit different protein expression profiles

To examine if there is a common pathway of regulation between the O and T phenotypes of the 3 pneumococcal strains, up to 20 protein spots which were substantially differentially regulated (with a minimum of 2-fold change between the two phenotypes) were subjected to identification by mass spectrometry. In this analysis, the proteins identified as up-regulated in the O variant of D39 include pyruvate oxidase (SpxB; GI Accession No. 15900627; 3-fold), and bifunctional acetaldehyde-CoA/alcohol dehydrogenase (GI Accession No. 15903879; 11-fold), whereas the protein identified as up-regulated in the T variant include glyceraldehyde-3-phosphate dehydrogenase (GAPDH; GI Accession No. 15901835; 16-fold) and lactate dehydrogenase [GI Accession No. 4138534; 3-fold] (Supplementary Tables S1 and S2). Generally, fewer proteins were significantly differentially expressed between the O and T variants of WCH16, compared to D39 variants. Notably, guanosine 5′-monophosphate oxidoreductase (GuaC; GI Accession No. 169833329; 4-fold) and maltose operon transcriptional repressor (GI Accession No. 15901927; 2-fold) were significantly up-regulated in the O variant of WCH16, while fructose-bisphosphate aldolase (Fba; GI Accession No. 15900513; 4-fold) and a branched chain amino acid transport ABC transporter substrate-binding protein (GI Accession No. 237650408; 3-fold) were significantly up-regulated in the T variant (Supplementary Tables S1 and S3). The significantly up-regulated proteins in the O variant of WCH43 include formate acetyltransferase (GI Accession No. 15900375; 6-fold), fructose-bisphosphate aldolase (Fba; GI Accession No. 15900513; 5-fold) and anaerobic ribonucleoside triphosphate reductase (NrdD; GI Accession No. 15900138; 7-fold). In the T variant, proteins identified as up-regulated include glyceraldehyde-3-phosphate dehydrogenase (GAPDH; GI Accession No. 15901835; 8-fold) and ribose-phosphate pyrophosphokinase (GI Accession No. 15899974; 7-fold) (Supplementary Tables S1 and S4).

**Figure 3:**
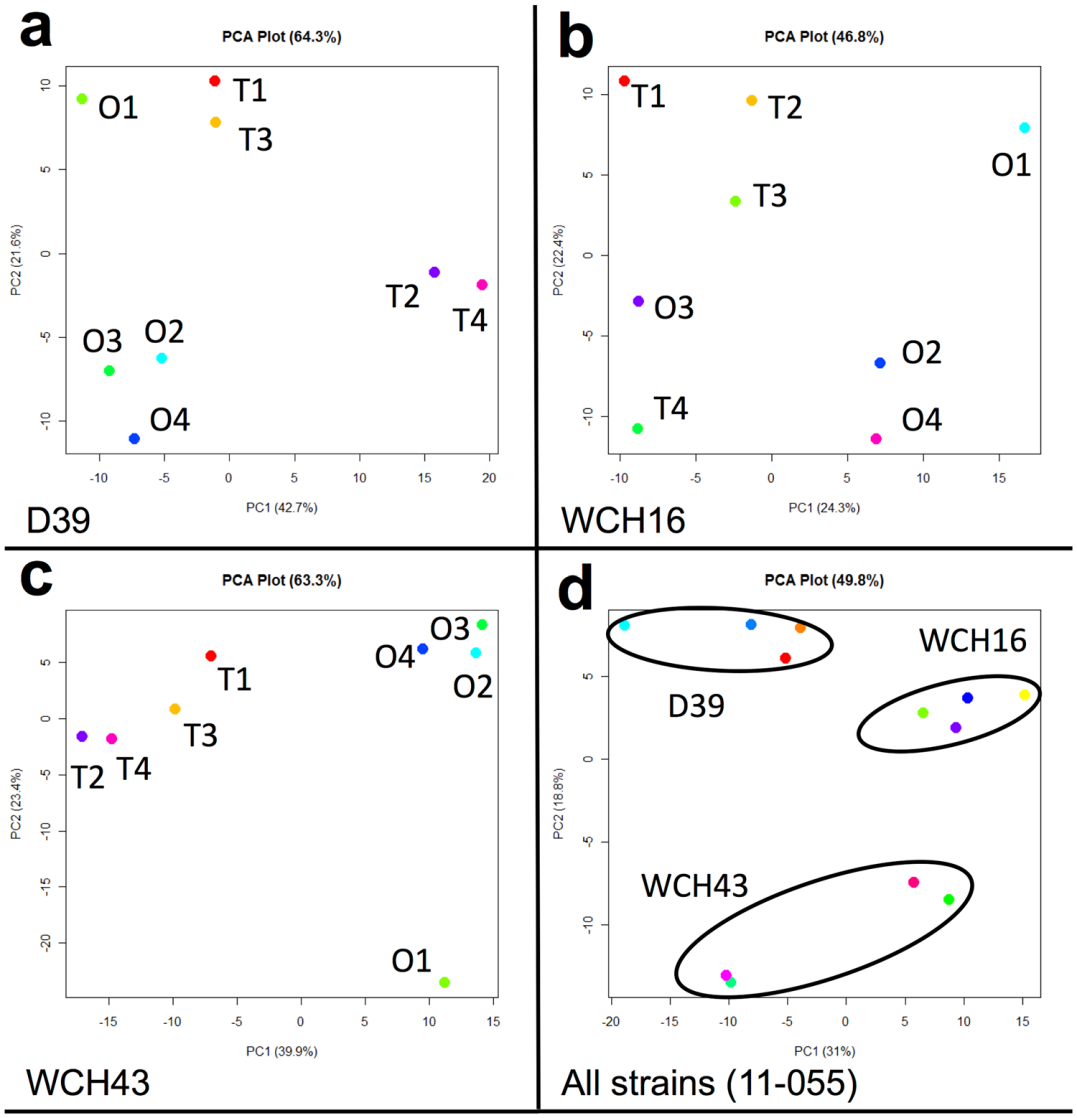
Principal component analysis of DIGE data from four independent experiments. (**a**) Transparent and opaque variants of strain D39 are separated in the first principal component (PC), representing 42.7% of overall variability of the dataset; (**b**) No linear separation of transparent and opaque variants of strain WCH16 in the first nine PCs (only the first two shown); (**c**) Separation of transparent and opaque variants of strain WCH43 in the first PC, representing 39.9% of overall variability of the data. (**d**) Data from DIGE experiment including all three strains. Clustering of strains can be observed, however only separation of WCH43 transparent and opaque strains in the first PC. Circles around different strains are arbitrary in their shape and size and are for visualization only.

In order to reduce the dimensionality of the 2D-DIGE data, we applied principal components analysis (PCA) to the protein expression patterns between the O and T variants of the 3 strains. Our PCA plots show that the O and T variants of D39 are separated in the first principal component (PC), which accounts for 42.7% of overall variability of the dataset (Fig. 3). However, there was no linear separation of the O and T variants of WCH16 in the first nine PCs, while separation of the O and T variants of WCH43 was observed in the first PC, accounting for 39.9% of overall variability of the data.

**Figure 4:**
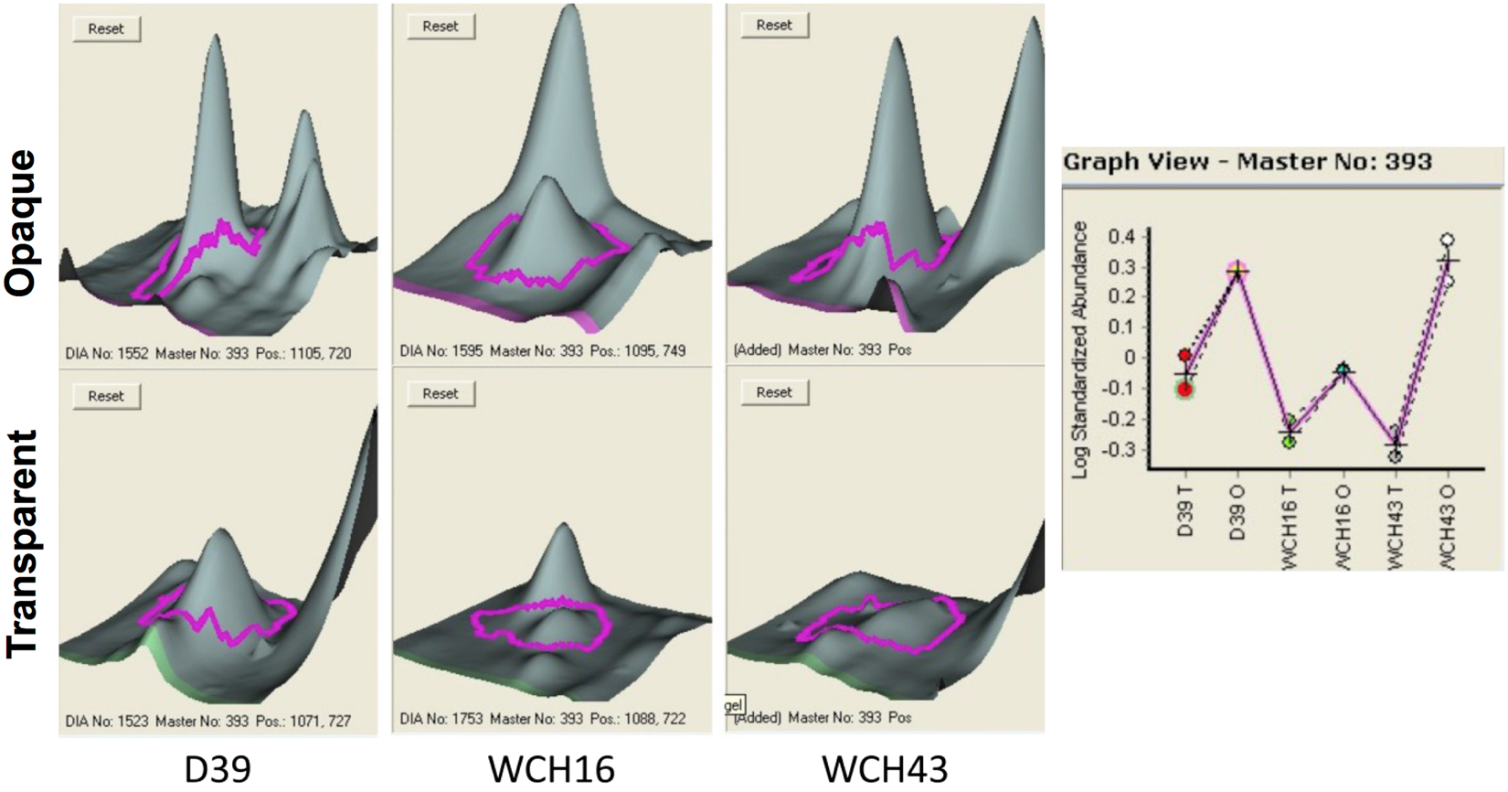
3D view of protein spot 393 in all three strains, upper view; opaque variant, lower view; transparent variant. Right side: Expression profile of spot 393 in all three strains.

As a direct comparison of the three independent DIGE experiments was not possible due to non-congruent spot patterns between D39, WCH16 and WCH43, a second DIGE experiment was conducted to interrogate for identical pattern of protein regulation (11-055). Only spot 393 was consistently statistically significantly regulated between the O and T phenotype in all three strains (Fig. 4). Proteins contained in this spot were identified by mass spectrometry as adenylosuccinate synthetase, UDP-N-acetylmuramate–L-alanine ligase, ATP-dependent Clp protease ATP-binding subunit, 30S ribosomal protein S1 and capsular polysaccharide biosynthesis protein Cps4J (Supplementary Table S1).

### SpxB analysis

**Figure 5:**
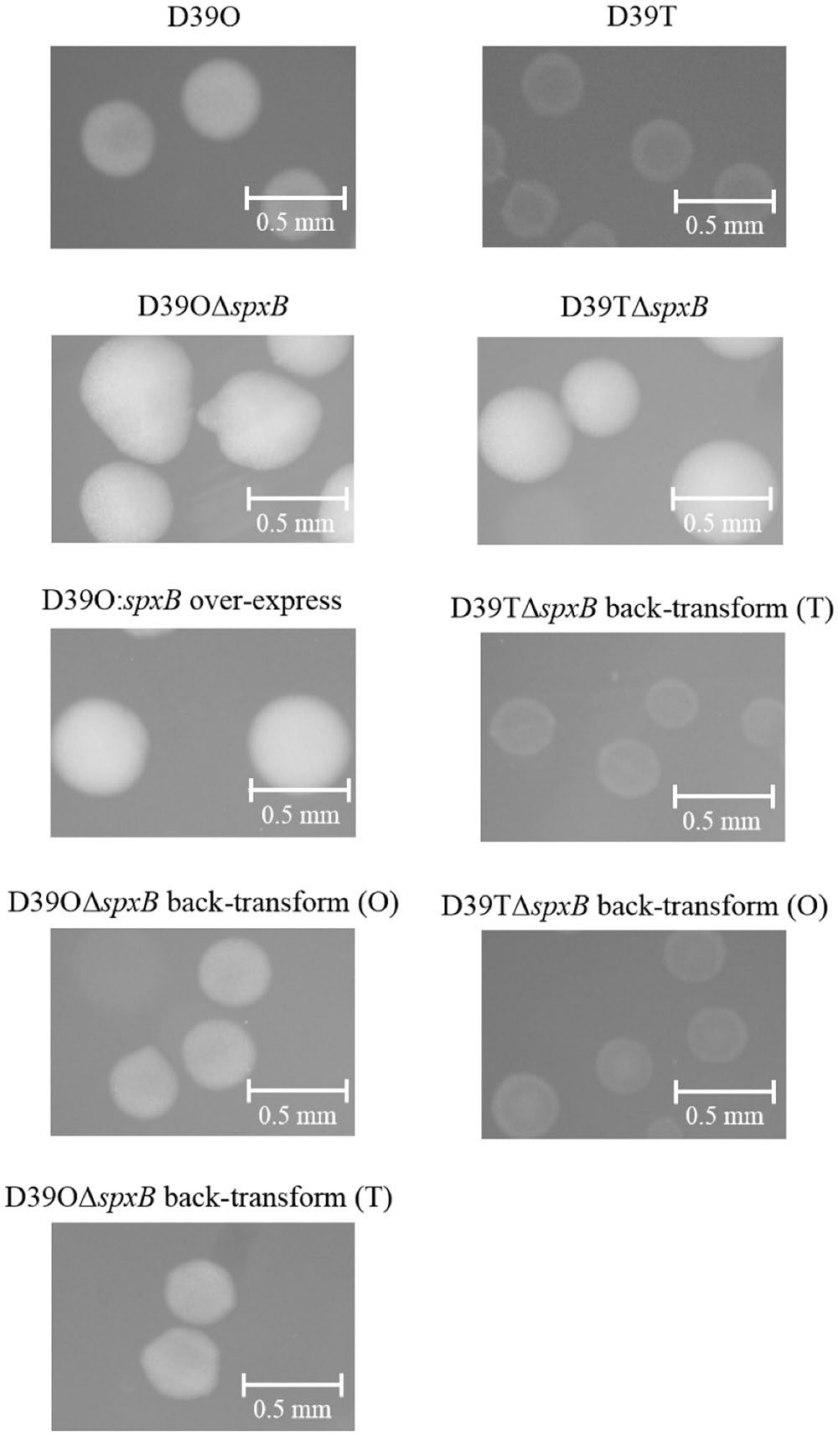
Colony morphology of various D39 SpxB mutants. Strains were streaked for single colonies on THY+ catalase agar plates and images were captured using Nikon SMZ1000 on a dissecting microscope and viewed through cellSens software. D39O, strain D39 opaque; D39T, strain D39 transparent; D39OΔ*spxB*, D39O *spxB* mutant; D39TΔ*spxB*, D39T *spxB* mutant; D39O:*spxB* over-express, D39O SpxB over-expression SpxB from D39T using a plasmid with constitutively expressing promoter; D39TΔ*spxB* back-transform (T), D39TΔ*spxB* with *spxB* from strain D39T re-inserted back; D39OΔ*spxB* back-transform (O), D39OΔ*spxB* with *spxB* from strain D39O re-inserted back; D39TΔ*spxB* back-transform (O), D39TΔ*spxB* with *spxB* from strain D39O re-inserted back; D39OΔ*spxB* back-transform (T), D39OΔ*spxB* with *spxB* from strain D39T re-inserted back.

**Figure 6:**
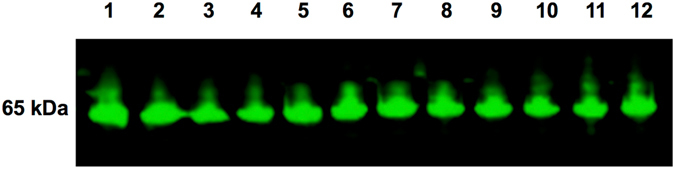
Quantitative Western blot analysis of phenotypic variants against anti-SpxB mouse anti-serum. Equal amount of whole cell lysates were added to each well. Two biological replicates of each strain and their variants were compared. Lanes 1–2, D39 opaque; lanes 3–4, D39 transparent; lanes 5–6, WCH16 opaque; lanes 7–8 WCH16 transparent; lanes 9–10, WCH43 opaque; lanes 11–12, WCH43 transparent.

A previous study had reported lower SpxB expression in O variants of *S*. *pneumoniae* serotypes 6B and 9 V^7^, which contrasts with our findings with the O variant of D39. Therefore, in order to further assess the impact of SpxB expression on colony opacity, the *spxB* gene was deleted from the O and T variants of D39 by targeted deletion replacement mutagenesis using overlap PCR^15^. Both O and T *spxB* mutant derivatives of D39 appeared larger, rounder and were more of O variant morphology on THY-catalase plates compared to D39O wild-type (D39O WT) (Fig. 5). Since the deletion of *spxB* in the variants appeared to produce markedly more O colonies, the *spxB* gene from D39O was cloned into plasmid pAL3 and used to transform D39O for ectopic expression. The resultant mutant, D39O-pAL3::*spxB*, had the same colony phenotype compared to D39O WT (Fig. 5). Interestingly, quantitative Western blotting of whole cell lysates from D39O, WCH16O, WCH43O and their respective T variants using mouse anti-SpxB serum showed no apparent difference in the amount of SpxB produced by any of the variants (Fig. 6).

### GAPDH activity is increased in T variants of D39 and WCH43

Our proteomic data showed that GAPDH was up-regulated in the T variant of D39 and up-regulated in 4 out of 5 GAPDH spots in the T variant of WCH43. However, GAPDH type 1 was up-regulated in the O variant of WCH16. In order to investigate these differences, the GAPDH activity between the O and T variants of D39, WCH16 and WCH43 were compared as described in Methods. For D39 and WCH43, the GAPDH activity in the T variant was significantly higher (about 1.5-fold) compared to its O counterpart, while there was no significant difference in GAPDH activity between the O and T variants of WCH16 (Table 1).

**Table 1:** GAPDH activity of pneumococcal variants.

Strain	GAPDH activity T/O ± SEM	*p*-value (*t*-test)
D39	1.50 ± 0.12	*p* < 0.001
WCH16	0.87 ± 0.17	ns
WCH43	1.49 ± 0.17	*p* < 0.05

The activity of three biological replicates of each strain (D39, WCH16 and WCH43) was calculated over the linear portion of the graph and expressed as a mean relative to the opaque variant of that strain.

### Quantitative Western Blot Analysis of Various Proteins shows no consistent regulation

**Figure 7:**
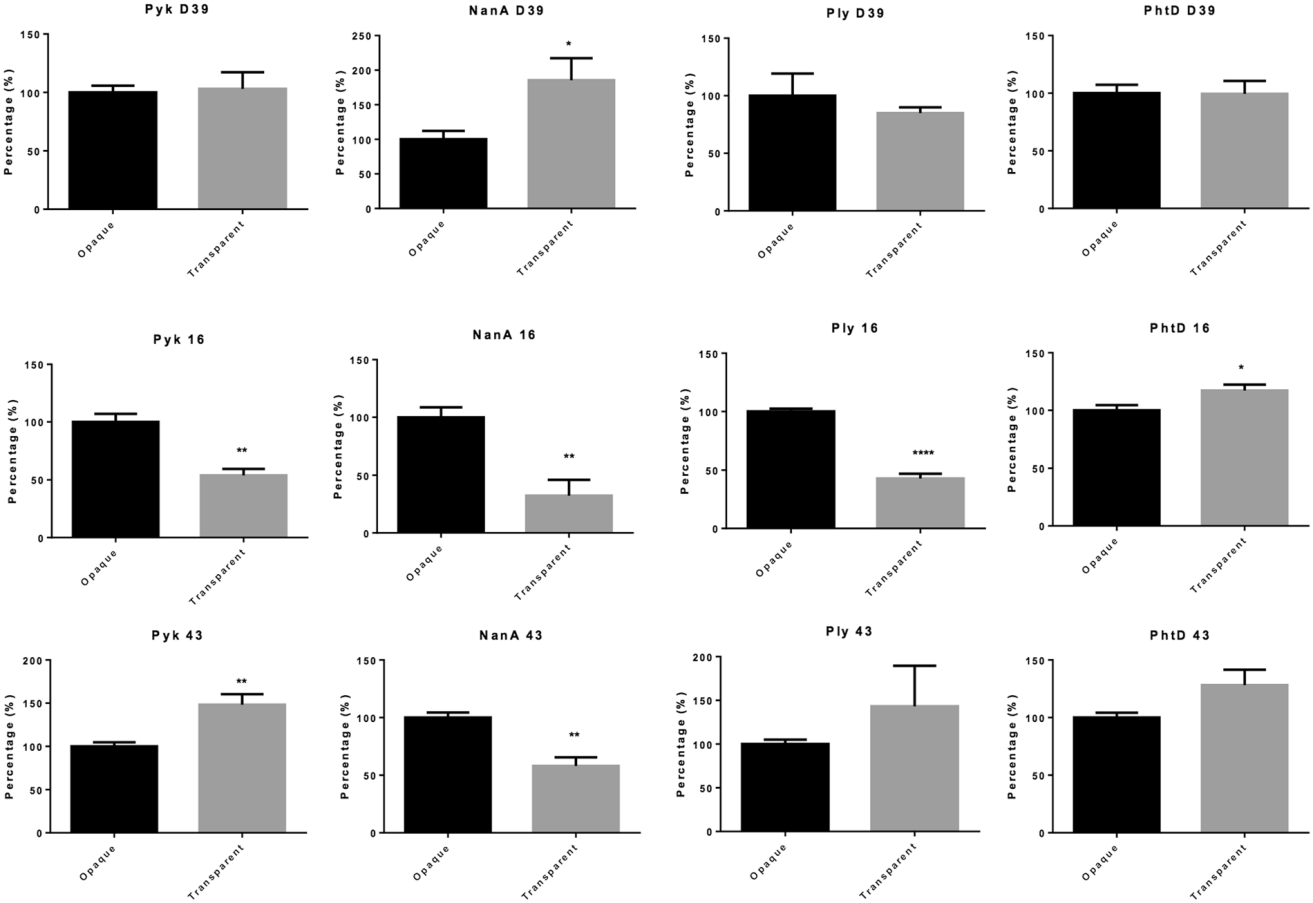
Quantitative Western blotting of pneumococcal proteins from lysates of opaque and transparent variants of D39, WCH16 and WCH43. Data are fluorescence intensity of the protein-reactive species, relative to that of the PsaA loading control for each strain (mean ± SEM of quadruplicate experiments). *P < 0.05; **P < 0.01; unpaired *t*-test, two-tailed.

It was reported previously that O and T variants express different amounts of certain pneumococcal virulence factors and proteins involved in metabolism^3, 7^. Therefore, the expression levels of 14 pneumococcal proteins of the 2 variants of D39, WCH16 and WCH43 was evaluated by quantitative Western blotting of cell lysates using protein-specific mouse polyclonal antisera. Of these, 4 proteins (pyruvate kinase (Pyk), neuraminidase A (NanA), pneumolysin (Ply) and pneumococcal histidine triad protein D (PhtD) showed significant differences in the expression levels between the 2 variants in at least one of the three strains (Fig. 7). Interestingly, none of the proteins was shown to be consistently up-regulated in a particular variant in all 3 strains, suggesting that these proteins could play distinct roles in the physiology of different pneumococcal strains.

## Discussion

The ability of respiratory pathogens such as *Haemophilus* *influenzae* and *Neisseria* *meningitidis* to disseminate from the nasopharynx to deeper host tissues to cause invasive disease has been shown to be dependent on their ability to reversibly adapt to changes in the microenvironment^16–19^. For *S*. *pneumoniae*, the phenomenon of opacity phase variation, first described over 2 decades ago^3^, was suggested to play a critical role in pneumococcal pathogenesis, adaptation to different host microenvironments^20^ as well as host-pathogen interaction. It was demonstrated (and also subsequently) that when apparently identical *S*. *pneumoniae* strains are isolated simultaneously from the nasopharynx and blood of patients with invasive disease, those from the nasopharynx are largely in the T phase, whilst those from the blood are almost exclusively in the O phase^3–5, 13^. While it has been demonstrated that the O variants express more capsule (and thus relatively less teichoic acid) than the T variant^3, 7^, phase variation could still be observed in unencapsulated pneumooccal mutants^3^. This suggests that factors other than capsule are likely to make an important contribution to this phenomenon.

Although the phenomenon of phase variation has been clearly demonstrated in *S*. *pneumoniae*, there is discordance in the literature regarding the contribution of protein expression patterns to colony opacity^3, 7, 11^. We reasoned that this could have been due to the fact that the pneumococcal strains and analytical techniques in each of those studies were quite different. Therefore, in order to gain further insight on the nature of proteins that might be central to the transition from nasopharygeal colonization to invasive disease, we have performed a detailed proteomic analysis of colony opacity phase variants in 3 *S*. *pneumoniae* strains with distinct pathogenicity characteristics (D39 [serotype 2], WCH16 [serotype 6A] and WCH43 [serotype 4]).

Cross comparisons of up to 20 up-regulated protein spots of O and T variants of the 3 strains show that there is very little overlap in the protein expression patterns between all 3 strains. The differentially-regulated proteins can generally be classified into five groups: those involved pyruvate metabolism, glycolysis to pyruvate production, transcription/translation proteins, and those required to maintain cellular health and sugar/amino acid transport. Most of the proteins involved in pyruvate metabolism and transcription/translation were found to be up-regulated in the O variants, whereas the ABC transporters and those involved in glycolysis up to pyruvate were upregulated in the T variants. Four proteins involved in pyruvate metabolism were identified in this study as being differentially expressed between O and T variants of D39 and WCH16 – SpxB (up-regulated in D39O vs. D39T); formate acetyltransferase (up-regulated in WCH16O vs. WCH16T); bifunctional acetaldehyde-CoA/alcohol dehydrogenase, AdhE (up-regulated in D39O vs. D39T); and lactate dehydrogenase, Ldh (up-regulated in WCH16T vs. WCH16O and in WCH43T vs. WCH43O).

Previous studies have shown that SpxB was differentially expressed protein between O and T variants of *S*. *pneumoniae* serotypes 4, 6B, 9 V and rough (unencapsulated) derivatives of D39^7, 21, 22^, and it was significantly up-regulated only in D39O *vs*. D39T in this study. A plausible explanation for the observed differential expression of SpxB could be that more cell death occurs in the T variant during growth to *A*_600_ = 0.5^23^. Cells at this density are in mid- to late-logarithmic growth phase whereby some SpxB is released into the medium, which would not be quantified from cell lysates used in the proteomic analysis. Interestingly, there was no detectable difference in SpxB expression between the O and T variants of any of the 3 strains by quantitative Western blotting. SpxB is known to exist in isoforms when run on a 2D-gel^24^, thus, if all the isoforms were quantified in all variants of the 3 strains, there may not be an overall significant change in SpxB expression. Other likely explanations for the discrepancies in SpxB expression by O and T variants between studies could be due to differences in the pneumococcal strain, growth media and cell density. For example, Overweg *et al*.^7^ used a serotype 9 V strain (strain p10) and grew the bacteria in THY to *A*_550_ = 0.3 (early to mid-logarithmic phase) compared to D39 (serotype 2), WCH43 (serotype 4) and WCH16 (serotype 6A) grown in C + Y to mid- to late-logarithmic phase in this study.

In order to further investigate the role of SpxB in pneumococcal phase variation, *spxB* was deleted in strain D39 which resulted in a larger and hyper-opaque phenotype as documented in other studies^11, 21, 25–27^. This, in part, could be attributed to an increase in capsule production^11^ or could be a consequence of reduced production of hydrogen peroxide and thus an increase in biomass production over time as a result of decreased cell death. However, it is unlikely that reduced SpxB expression is the switch that determines pneumococcal opacity phenotypic variation, as ectopic over-expression of SpxB in the O variant did not result in a T phenotype. Furthermore, when the *spxB* gene from either O or T variant was transformed into D39O and D39T *spxB* mutants, the transformants reverted back to their respective WT phenotypes, regardless of the origin of the *spxB* gene. This suggests that the *spxB* gene itself is not responsible for the change in pneumococcal colony opacity variation, but rather, it can act in concert with other proteins to generate the different colony opacity phenotypes.

Our proteomic analysis also revealed that GAPDH was significantly up-regulated in the T variants of D39 and WCH43, but not in WCH16, and this was confirmed by GAPDH activity assays. This would suggest that in the T variants, increase in GAPDH activity results in carbon metabolic flux being directed to the production of pyruvate and generation of ATP during glycolysis. Consistent with this is a significant increase in expression of Ldh (which converts pyruvate to lactate) in the T variants of the 3 strains, confirming a role for this enzyme in maintenance of redox balance in pneumococcal central metabolism^28^.

## Conclusions

The findings of the proteomic analysis of the O and T variants of 3 pneumococcal strains examined in this study suggest that a combination of metabolic activities and overall protein expression patterns contribute to the O and T phenotypes, and that these opacity variations are likely strain dependent. Thus, it is unlikely that there is one single protein that switches the pneumococcus from O variant to T variant, and vice versa. Rather, it suggests that opacity phase variation in *S*. *pneumoniae* is complex and multifactorial, requiring a combination of factors and events that act together to produce a certain opacity phenotype that contributes to its pathogenicity characteristics. For example, *in vivo*, D39 and WCH43 are more likely to cause bacteremia where the O variant is more commonly isolated, while WCH16 is more adept at colonizing the nasopharynx, a niche where T variants are more commonly isolated. This might explain why the protein expression profiles of D39 and WCH43 are more similar to each other than to that of WCH16. The proteins that are up-regulated in the T variant, such as PhtD, appear to be those involved in adherence, while those up-regulated in the O variant include stress proteins, those that are associated with virulence, such as PurA, or those required for repair of the cell as it encounters more stresses during invasion, such as host immune cells present in the blood.

## Methods

### Bacterial strains and growth conditions

The pneumococcal strains used in this study are serotype 2 (D39; Sequence Type [ST] 595), serotype 4 (WCH43; ST205), and serotype 6A (WCH16; ST4966). Serotype-specific capsule production was confirmed by Quellung reaction, as described previously^29^. Pure opaque (“O”) and transparent (“T”) phase variants from minimally passaged frozen stocks of the three strains were selected after growth on Todd-Hewitt broth supplemented with 1% yeast extract (THY)-catalase plates and observed under oblique, transmitted light as described previously^3^. Aliqouts of each phenotypic variant of all strains were frozen at −80 °C and confirmed to be pure by several *in vitro* passages on THY-catalase plates; these served as working stocks for subsequent experiments. Cell pellets were prepared by growing the strains in C + Y broth^30^ to *A*_600_ = 0.5 approx. 1 × 10^8^ colony-forming units [CFUs], an aliquot was plated on THY-catalase plate to confirm purity, while the rest of the culture was centrifuged at 10,000 ×  *g* for 10 min and the pellet frozen at −80°C until required. For proteomic analyses, each phenotypic variant of each strain was grown on four separate occasions to obtain quadruplicate samples for analysis. Samples were lysed in protease inhibitor cocktail tablet [Roche Diagnostic], 1 μg/ml DNase I [Roche], 1 μg/ml RNase A [Roche] and 100 U/ml mutanolysin [Sigma] and disrupted using an Aminco French Pressure Cell at 12,000 psi. Lysates of O and T variants of strains D39, WCH16 and WCH43 were separated into membrane and cytosolic fractions by ultracentrifugation at 255,000 × *g* for 1 hour at 4 °C.

### 2D-DIGE Procedures

#### Chemicals and Solvents

Urea, ammonium bicarbonate and acetonitrile (ACN) were obtained from Merck (Darmstadt, Germany); thiourea, CyDyes, iodoacetamide (IAA) and Pharmalyte 3–10 were obtained from GE Healthcare (Little Chalfont, UK); 3-[(3-cholamidopropyl) dimethylammonio]-1-propanesulfonate (CHAPS) was purchased from Roche Diagnostics (Basel, Switzerland). The EZQ protein quantification assay was from Life Technologies (Carlsbad, USA). Equilibration buffer and Electrode solutions for SDS-PAGE were obtained from Serva (Heidelberg, Germany). Dithiothreitol (DTT), Hydroxyethyldisulfide (HED), dimethylformamide (DMF), formic acid and L-lysine were purchased from Sigma-Aldrich (St. Louis, USA). The ReadyPrep 2D clean up kit was obtained from Bio-Rad (Hercules, USA). Sequencing grade modified trypsin was purchased from Promega (Finchburg, USA). All buffers were prepared using ultra-pure water from a Thermo Fisher Scientific system (Waltham, USA).

#### DIGE labelling

Two sets of DIGE experiments were performed. The first set consists of three independent DIGE experiments using O and T variants of strains D39, WCH16 and WCH43, respectively (Supplementary Table S5). The second set used the variants of all the 3 strains in a combined DIGE approach (Supplementary Table S5). The three powdered CyDyes were resuspended in DMF, aliquoted in 1 μl volumes (200 pmol CyDye) and stored under argon at −80 °C until required. Before labelling, equal protein amounts of the respective cytosolic and membrane fractions were mixed. Thereafter, 200 pmol of either Cy3 or Cy5 dye was added to the individual mixtures of cytosolic and membrane fractions. The internal pooled standard (IPS) was prepared by pooling equal protein amounts of each sample applied in this study and labelled with 200 pmol Cy2 dye per 100 μg of protein. After labelling, DTT solution (0.167 g DTT/100 μl H_2_O) and Pharmalyte 3–10 were added to each sample, both to an individual final volume of 2% (v/v). The samples were then subjected to isoelectric focusing.

#### Isoelectric focusing (IEF)

IPG strips with a non-linear pH gradient from 3–11 and a length of 24 cm (GE Healthcare) were rehydrated overnight in 450 μl TUC1% (6 M Urea, 2 M Thiourea, 50% ACN, 1% CHAPS, 0.5% Pharmalyte 3–10, 200 mM HED), samples were applied to the IPG strips via anodic cup-loading and IEF was performed according to published protocols^31^ on a IPGphor II (GE Healthcare) in darkness. The IEF was stopped after 27,000 Vhrs.

#### SDS-PAGE

The IPG strips were equilibrated in the dark according to the protocol of Serva. Electrophoresis was carried out on T = 12.5% precast polyacrylamide gels (Serva) using an Ettan DALT 12 electrophoresis unit (GE Healthcare) according to the protocol of Serva. For the independent DIGE experiments of D39, WCH16 and WCH43, SDS-PAGE in the DIGE experiment combining all variants was carried out on T = 12.5% precast polyacrylamide flatbed gels (Serva) using the HPE FlatTop Tower unit (Serva) according to the manufacturers protocol.

#### DIGE Imaging and analysis

SDS-PAGE gels were scanned using an Ettan DIGE Imager (GE Healthcare) with a resolution of 100 μm. The exposure times of the individual channels (Cy2, Cy3 and Cy5) were set to yield a maximum of approximately 35,000 intensity units. The resulting images were horizontally flipped before image analysis using ImageQuant TL (Version 7.0, GE Healthcare).

Image analysis was undertaken using DeCyder 2D software (version 7, GE Healthcare). Each gel image was processed separately in the Differential In-gel Analysis (DIA) module of DeCyder prior to export to the Biological Variation Analysis (BVA) module. In all DIGE experiments, protein expression in the T variant of every strain was subjected to statistical comparison with its O counterpart (D39O vs. D39T; WCH16O vs. WCH16T; WCH43O vs. WCH43T) to detect spots that are differentially expressed using unpaired two-tailed Students *t*-test. Those spots that returned a *p*-value of <0.05 were accepted. For the second DIGE experiments, spots with a significant *p*-value were further verified to exhibit a consistent regulation pattern, i.e. up/down regulated in T vs. O in all three strains.

#### Protein identification

For the first DIGE set, 500 μg of protein consisting of equal amounts of the cytosolic and membrane fractions of the respective T and O variant of each strain were pooled and separated according to the protocols described above (omitting the labelling reaction). After SDS-PAGE, the gels were fixed using 40% ethanol and 10% acetic acid and proteins stained afterwards using Coomassie brilliant blue. The proteins of interest were picked manually, ensuring the correct spot identity by comparison of the DIGE derived spot pattern with the spot pattern on the Coomassie brilliant blue stained gel. Liquid chromatography electrospray ionisation ion-trap mass spectrometry (LC-ESI-IT MS) using an HTC Ultra 3D ion trap (Bruker Daltonics) was performed as detailed in Supplementary Information. For the second DIGE set of experiments, the proteins of interest were excised from the DIGE gels using an Ettan Spot Picker (GE Healthcare). To account for the lower protein loading in the DIGE gels relative to that of the Coomassie stained gels, the proteins were identified using a LTQ Orbitrap mass spectrometer (Thermo Fisher). Liquid chromatography-mass spectrometry (LC-MS) with the Orbitrap was performed using a Shimadzu Prominence LC-20AD nano HPLC (Shimadzu, Japan) and Mass Spectrometer, coupled using the Nanospray Source I (Thermo Fisher Scientific) and a nanospray emitter (NewObjective, MA).

#### Mass spectrometry data analysis

For HTC ion trap MS, DataAnalysis (Version 3.4, Bruker Daltonics) was used to perform peak detection and de-convolution of the MS and MS/MS spectra. The derived compound lists were exported into BioTools (Version 3.1, Bruker Daltonics) then submitted to Mascot (Version 2.2, Matrix Science). For LTQ Orbitrap MS, data analysis was performed using the XCalibur software (Version 2.0.7, Thermo Fisher Scientific). MS/MS spectra were extracted and submitted to the Mascot search engine using Proteome Discoverer (Version 1.3, Thermo Fisher Scientific).

### Over-expression of SpxB

The overexpression of *spxB* in the O variant of D39 was achieved by cloning the *spxB* gene from the T variant of D39 into a modified *Streptococcus*-*Escherichia coli* shuttle vector pVA838 [pAL3]^32^, which had been engineered to express *spxB* genes under the *S*. *pneumoniae* aminopterin resistance operon (*ami*) promoter^33^. To generate *spxB*-expressing plasmid (pAL3:*spxB*), *spxB*-specific forward (5′-TCCAATTCTATGTAATCGAATTCTCCAAG-3′) and reverse (5′-GAAAATCAAAGAATGAATTCTACAAGTTTC-3′) primer sequences carrying *Eco*RI sites were used to PCR-amplify *spxB* (1.8 kb) from a D39T DNA template, and cloned into the corresponding *Eco*RI-digested and dephosphorylated site in pAL3. The ligation mixture was initially used to transform competent *E*. *coli* XL-10 after which the recombinant plasmid was extracted and confirmed to be of the right size and orientation. The resultant pAL3:*spxB* clone was then used to transform the O variant of D39 essentially as described previously^32^.

### GAPDH activity assays

In order to measure the GAPDH activity of the various O and T variants cell pellets were resuspended in 1 ml PBS, disrupted by sonication and clarified at 13,000 ×  *g* for 10 min at 4 °C. Extracts were kept on ice until required. The GAPDH assay was a measurement of the reduction of NAD (β-Nicotinamide adenine dinucleotide hydrate) according to Fillinger *et al*.^34^, with modifications. Using a 1 ml disposable cuvette, 900 μl triethanolamine/sodium arsenate buffer (125 mM triethanolamine [Sigma], 5 mM L-cysteine [Sigma], 20 mM sodium arsenate [Sigma], 50 mM disodium hydrogen phosphate, Na_2_HPO_4_ [Merck], pH 9.2), 2 mM NAD [Sigma] and 60 μl cell extracts were mixed and read at *A*_340nm_ at 25 °C. Initially, the mixture was allowed to react for about 10 seconds to ensure that there is nothing in the solution reducing the NAD. Thereafter, 4 mM D-glyceraldehyde-3-phosphate, G-3-P (substrate) [Sigma] was added and the reaction was recorded for 3–5 minutes. One unit causes an initial reaction of reduction of one micromole of NAD per minute and is calculated as follows, where the Δ*A*_340_/minute is the linear portion of the graph:

$${Units}\,{per}\,{mg}=\frac{{\rm{\Delta }}A340\,{\rm{nm}}\,{\rm{per}}\,{\rm{minute}}}{6.22\times {\rm{mg}}\,{\rm{enzyme}}\,{\rm{per}}\,{\rm{ml}}\,{\rm{reaction}}\,{\rm{mixture}}}$$

The amount of GAPDH was then calculated relative to the O variant of each strain. Data were analysed using Student’s unpaired *t*-test.

### Quantitative Western blotting

Relative protein expression was determined by quantitative Western blotting of whole cell lysates of *S*. *pneumoniae* O and T variants and their recombinant derivatives. Briefly, pneumococci were grown to *A*_600nm_ = 0.19 in SB or *A*_600nm_ = 0.5 in C + Y, concentrated 40-fold and lysed by treatment with 0.1% [w/v] DOC. The total protein in the lysates was determined using the BCA Protein Assay kit [Thermo Scientific]. Approximately 15 μg of the lysates were resuspended in lysate buffer (2.5% [v/v] β-mercaptoethanol, 31.25 mM Tris-HCl, 1% [w/v] DOC, 5% [v/v] glycerol, and 0.025% [w/v] bromophenol blue; pH 6.8) and subjected to SDS-PAGE. Separated proteins were electro-blotted onto nitrocellulose membrane [Invitrogen] using the iBlot Dry Blotting System according to the manufacturer’s instructions [Invitrogen], and then probed with a 1:5,000 dilution of target mouse-polyclonal antiserum (primary antibody) in assay buffer (Tris buffered saline containing 0.02% Tween 20 [TTBS]). After extensive washing TTBS, the blot was reacted with donkey anti-mouse IRDye 800 CW secondary antibody [LI-COR Biosciences, Nebraska, USA] at a dilution of 1:50,000. The blot was scanned using the Odyssey Infrared Imaging System [Li-COR Biosciences]. Relative quantification of protein expression between the strains was performed using the Odyssey Infrared Imaging System application software v 3.0.21 by comparing units of fluorescence [LI-COR Biosciences]. Purified recombinant protein was used as a marker for each protein of interest, and reactivity to recombinant PsaA was used as a loading control.

## Electronic supplementary material

Supplementary Information

Dataset 1
