# Supplementary material for: Proteomic comparisons of opaque and transparent variants of Streptococcus pneumoniae by two dimensional-differential gel electrophoresis
Source: Sci Rep. 2017 May 26;7:2453. doi: 10.1038/s41598-017-02465-x (PMC5446427; doi:10.1038/s41598-017-02465-x)
Supplement: Supplementary file 1 — Supplementary Information [file 41598_2017_2465_MOESM1_ESM.pdf]

Proteomic comparisons of opaque and transparent variants of *Streptococcus pneumoniae* by two dimensional-differential gel electrophoresis

Melissa H. Chai[1¶] Florian Weiland[2,3¶††], Richard M. Harvey[1], Peter Hoffmann[2,3†], Abiodun D. Ogunniyi[1†§*], & James C Paton[1†]

[1] Research Centre for Infectious Diseases, School of Biological Sciences, The University of Adelaide, South Australia 5005, Australia

[2] Adelaide Proteomics Centre, School of Biological Sciences, The University of Adelaide, South Australia 5005, Australia

[3] Institute for Photonics and Advanced Sensing (IPAS), The University of Adelaide, South Australia 5005, Australia

[††]Present Address: Medical Research Council Protein Phosphorylation and Ubiquitylation Unit, School of Life Sciences, University of Dundee, Dundee DD1 5EH, United Kingdom

[§]Present Address: Australian Centre for Antimicrobial Resistance Ecology, School of Animal and Veterinary Sciences, The University of Adelaide, South Australia 5371, Australia

[¶]Co-first author; †Co-senior author; *For correspondence: david.ogunniyi@adelaide.edu.au

SUPPLEMENTARY METHODS

2D-DIGE Procedures

Chemicals and Solvents. Urea, ammonium bicarbonate and acetonitrile (ACN) were obtained from Merck (Darmstadt, Germany); thiourea, CyDyes, iodoacetamide (IAA) and Pharmalyte 3-10 were obtained from GE Healthcare (Little Chalfont, UK); 3-[(3-cholamidopropyl) dimethylammonio]-1-propanesulfonate (CHAPS) was purchased from Roche Diagnostics (Basel, Switzerland). The EZQ protein quantification assay was from Life Technologies (Carlsbad, USA). Equilibration buffer and Electrode solutions for SDS-PAGE were obtained from Serva (Heidelberg, Germany). Dithiothreitol (DTT), Hydroxyethyldisulfide (HED), dimethylformamide (DMF), formic acid and L-lysine were purchased from Sigma-Aldrich (St. Louis, USA). The ReadyPrep 2D clean up kit was obtained from Bio-Rad (Hercules, USA). Sequencing grade modified trypsin was purchased from Promega (Finchburg, USA). All buffers were prepared using ultra-pure water from a Thermo Fisher Scientific system (Waltham, USA).

Sample Preparation- Membrane protein fraction. The cell membrane pellet was rinsed briefly with *aqua bidest*, the supernatant was removed and the pellet was resuspended in TUX 4% buffer (7 M Urea, 2 M Thiourea, 50% Acetonitrile, 4% CHAPS). The mixture was sonicated 3 x 5 minutes in an ice-containing waterbath to dissolve the proteins. Still visible pellet remains were crushed mechanically using a pipette tip, the solution was vortexed thoroughly until it

appeared homogenous and stored at -20°C until further use. Protein concentrations of the membrane fractions were measured using an EZQ assay.

Sample Preparation- Cytosolic protein fraction. Approximately 5 ml of the cytosolic protein fraction was concentrated to a volume of 200 μl using Vivaspin 6 columns (GE Healthcare). Afterwards the proteins were precipitated using a ReadyPrep 2D clean up kit (Bio-Rad) according to the manufacturers protocol and the proteins were resuspended in TUX 4% buffer. The samples were stored at -20°C until further use. The protein concentration of the samples was estimated using an EZQ assay.

DIGE labelling. Two sets of DIGE experiments were performed. The first set consists of three independent DIGE experiments using O and T variants of strains D39, WCH16 and WCH43, respectively (Table S1). The second set used the variants of all the 3 strains in a combined DIGE approach (Table S1). The three powdered CyDyes were resuspended in DMF, aliquoted in 1 μl volumes (200 pmol CyDye) and stored under argon at -80°C until required. Before labelling, equal protein amounts of the respective cytosolic and membrane fractions were mixed. Thereafter, 200 pmol of either Cy3 or Cy5 dye was added to the individual mixtures of cytosolic and membrane fractions. The internal pooled standard (IPS) was prepared by pooling equal protein amounts of each sample applied in this study and labelled with 200 pmol Cy2 dye per 100 μg of protein. After labelling, DTT solution (0.167 g DTT/ 100 μl H_2O) and Pharmalyte 3-10 were added to each sample, both to an individual final volume of 2% (v/v). The samples were then subjected to isoelectric focusing.

Isoelectric focusing (IEF). IPG strips with a non-linear pH gradient from 3-11 and a length of 24 cm (GE Healthcare) were rehydrated overnight in 450 μl TUC1% (6M Urea, 2M Thiourea, 50% ACN, 1% CHAPS, 0.5% Pharmalyte 3-10, 200 mM HED), samples were applied

to the IPG strips via anodic cup-loading and IEF was performed according to published protocols [1] on a IPGphor II (GE Healthcare) in darkness. The IEF was stopped after 27,000 Vhrs.

SDS-PAGE. The IPG strips were equilibrated in the dark according to the protocol of Serva. Electrophoresis was carried out on T=12.5% precast polyacrylamide gels (Serva) using an Ettan DALT 12 electrophoresis unit (GE Healthcare) according to the protocol of Serva. For the independent DIGE experiments of D39, WCH16 and WCH43, SDS-PAGE in the DIGE experiment combining all variants was carried out on T=12.5% precast polyacrylamide flatbed gels (Serva) using the HPE FlatTop Tower unit (Serva) according to the manufacturers protocol.

DIGE Imaging. SDS-PAGE gels were scanned using an Ettan DIGE Imager (GE Healthcare) with a resolution of 100 μm. The exposure times of the individual channels (Cy2, Cy3 and Cy5) were set to yield a maximum of approximately 35,000 intensity units. The resulting images were horizontally flipped before image analysis using ImageQuant TL (Version 7.0, GE Healthcare).

Image Analysis. Image analysis was undertaken using DeCyder 2D software (version 7, GE Healthcare). Each gel image was processed separately in the Differential In-gel Analysis (DIA) module of DeCyder prior to export to the Biological Variation Analysis (BVA) module. In the DIA module, spot detection was performed based on an estimated 5,000 spots. Exclusion filters were set to reject spots with a slope of >1.1, an area of <300, a volume of <30,000 and a peak height of <80 or >65534. The resulting spot maps were inspected manually and regions that showed poorly resolved spot patterns were excluded from further processing (e.g., at the edges of the gels or areas affected by vertical streaking). The DIA workspace was then imported into BVA for spot matching and comparative analysis. To aid in the spot matching process, a several spots from different areas across the gels were matched manually to provide “landmarks”. The

automatic matching function including warping was then applied and the results were evaluated. Any errors in the automatically assigned spot matches were re-matched manually. In all DIGE experiments, protein expression in the T variant of every strain was subjected to statistical comparison with its O counterpart (D39O vs. D39T; WCH16O vs. WCH16T; WCH43O vs. WCH43T) to detect spots that are differentially expressed using unpaired two-tailed Students *t*-test. Those spots that returned a *p*-value of < 0.05 were accepted. For the second DIGE experiments, spots with a significant *p*-value were further verified to exhibit a consistent regulation pattern, i.e. up/down regulated in T vs. O in all three strains. Afterwards, the normalized spot volume of the respective dataset was exported in a tab separated text file using the XML Toolbox sub-program of DeCyder. Spot data in this text file was analyzed for q-values according to the equations of Storey and Tibshirani [2] as described earlier [3]. *Posthoc* power calculations were performed essentially as described previously [4]. This workflow was repeated for all three datasets.

Protein identification. For the first DIGE set, 500 μg of protein consisting of equal amounts of the cytosolic and membrane fractions of the respective T and O variant of each strain were pooled and separated according to the protocols described above (omitting the labelling reaction). After SDS-PAGE, the gels were fixed using 40% ethanol and 10% acetic acid and proteins stained afterwards using Coomassie brilliant blue. The proteins of interest were picked manually, ensuring the correct spot identity by comparison of the DIGE derived spot pattern with the spot pattern on the Coomassie brilliant blue stained gel. Spots were picked using individual pipette tips cut to the diameter of the spot size on the Coomassie brilliant blue stained gel. Liquid chromatography electrospray ionisation ion-trap mass spectrometry (LC-ESI-IT MS) using an HTC Ultra 3D ion trap (Bruker Daltonics) was performed as follows, with a preceding destaining

step to remove the Coomassie brilliant blue stain. An 1100 series HPLC system (Agilent Technologies) was coupled online to the mass spectrometer. The LC system was interfaced to the MS using an Agilent Technologies Chip Cube operating with a ProtID-Chip-150 (II), which integrates the enrichment column (Zorbax 300SB-C18, 4 mm, 40 nL), analytical column (Zorbax 300 SB-C18, 150 mm 75 m), and nanospray emitter. Two microlitres of sample was loaded on the enrichment column at a flow rate of 4 μL/min in Mobile Phase A (0.1% FA in 2% v/v ACN) and resolved with 1-30% gradient of Mobile Phase B (0.1% FA in 98% w/v ACN) over 32 minutes at 300 nL/min. Ionizable species (300 < m/z < 1,200) were trapped and the two most intense ions eluting at the time were fragmented by collision-induced dissociation. Active exclusion was used to exclude a precursor ion for 30 seconds following the acquisition of two spectra. For the second DIGE set of experiments, the proteins of interest were excised from the DIGE gels using an Ettan Spot Picker (GE Healthcare). To account for the lower protein loading in the DIGE gels relative to that of the Coomassie stained gels, the proteins were identified using a LTQ Orbitrap mass spectrometer (Thermo Fisher). LC-MS with the Orbitrap was performed using a Shimadzu Prominence LC-20AD nano HPLC (Shimadzu, Japan) and Mass Spectrometer, coupled using the Nanospray Source I (Thermo Fisher Scientific) and a nanospray emitter (NewObjective, MA). 5 μl from spot 393 was loaded onto an Acclaim PepMap100, C18, 5 µm, 100 Å, 300 µm i.d. x 5 mm (Dionex) enrichment column at a flow rate of 4 μL/min in Mobile Phase A (0.1% FA in 2% v/v ACN) and resolved over an Acclaim PepMap100, C18, 3 µm, 100 Å, 75 µm i.d. × 15 cm (Dionex) analytical column at 300 nL/min, 4-55% B gradient of Mobile Phase B (0.1% FA in 80% w/v ACN) over 30 minutes. Ionisable species (300 < m/z < 2000) were trapped and the six most intense ions eluting at the time were individually selected

for fragmentation by collision-induced dissociation. Active exclusion was used to exclude an already fragmented precursor ion from selection for a period of 15 seconds.

Protein identification. Mass spectrometry data analysis– For HTC ion trap MS, DataAnalysis (Version 3.4, Bruker Daltonics) was used to perform peak detection and de-convolution of the MS and MS/MS spectra. The derived compound lists were exported into BioTools (Version 3.1, Bruker Daltonics) then submitted to Mascot (Version 2.2, Matrix Science). For LTQ Orbitrap MS, data analysis was performed using the XCalibur software (Version 2.0.7,Thermo Fisher Scientific). MS/MS spectra were extracted and submitted to the Mascot search engine using Proteome Discoverer (Version 1.3,Thermo Fisher Scientific). In both cases, a maximum of two missed cleavages was allowed, carbamidomethylation of cysteine was set as a fixed modification and oxidation of methionine was set as a variable modification. On MS level, a deviation of 0.3 Da (HTC ion trap) or 20 ppm (LTQ Orbitrap) for the monoisotopic mass was tolerated; on MS/MS level a mass tolerance of 0.4 Da (HTC ion trap) or 0.8 Da (LTQ Orbitrap) for the monoisotopic mass was set. The resulting peak lists were searched against the NCBInr database 20130423, *Streptococcus pneumoniae* and mammalian taxonomy (1,476,058 sequences). Proteins were considered as identified if they had at least two matching unique peptides with an individual ion score above the homology threshold. If no homology threshold could be derived by MASCOT, the identity threshold was used instead.

Supplementary Information:

Table S1 includes the combined protein identifications of spots picked from the DIGE gels; Tables S2, S3 and S4 includes data for the standardized abundance and fold change values of protein spots for D39T *vs* D39O, WCH16T *vs* WCH16O, and WCH43T *vs* WCH43O, respectively; Table S5 contains the design of the DIGE experiments.

REFERENCES

1 Gorg, A., Drews, O., Luck, C., Weiland, F. & Weiss, W. 2-DE with IPGs. *Electrophoresis* **30 Suppl 1**, S122-132, doi:10.1002/elps.200900051 (2009).

2 Storey, J. D. & Tibshirani, R. Statistical significance for genomewide studies. *Proc Natl Acad Sci U S A* **100**, 9440-9445, doi:10.1073/pnas.1530509100 (2003).

3 Penno, M. A. *et al*. 2D-DIGE analysis of sera from transgenic mouse models reveals novel candidate protein biomarkers for human gastric cancer. *J Proteomics* **77**, 40-58, doi:10.1016/j.jprot.2012.07.002 (2012).

4 Penno, M. A., Bacic, A., Colegate, S. M., Hoffmann, P. & Michalski, W. P. Identifying candidate serum biomarkers of exposure to tunicamycins in rats using two-dimensional electrophoresis. *J Proteome Res* **8**, 2812-2826, doi:10.1021/pr801111a (2009).

D39

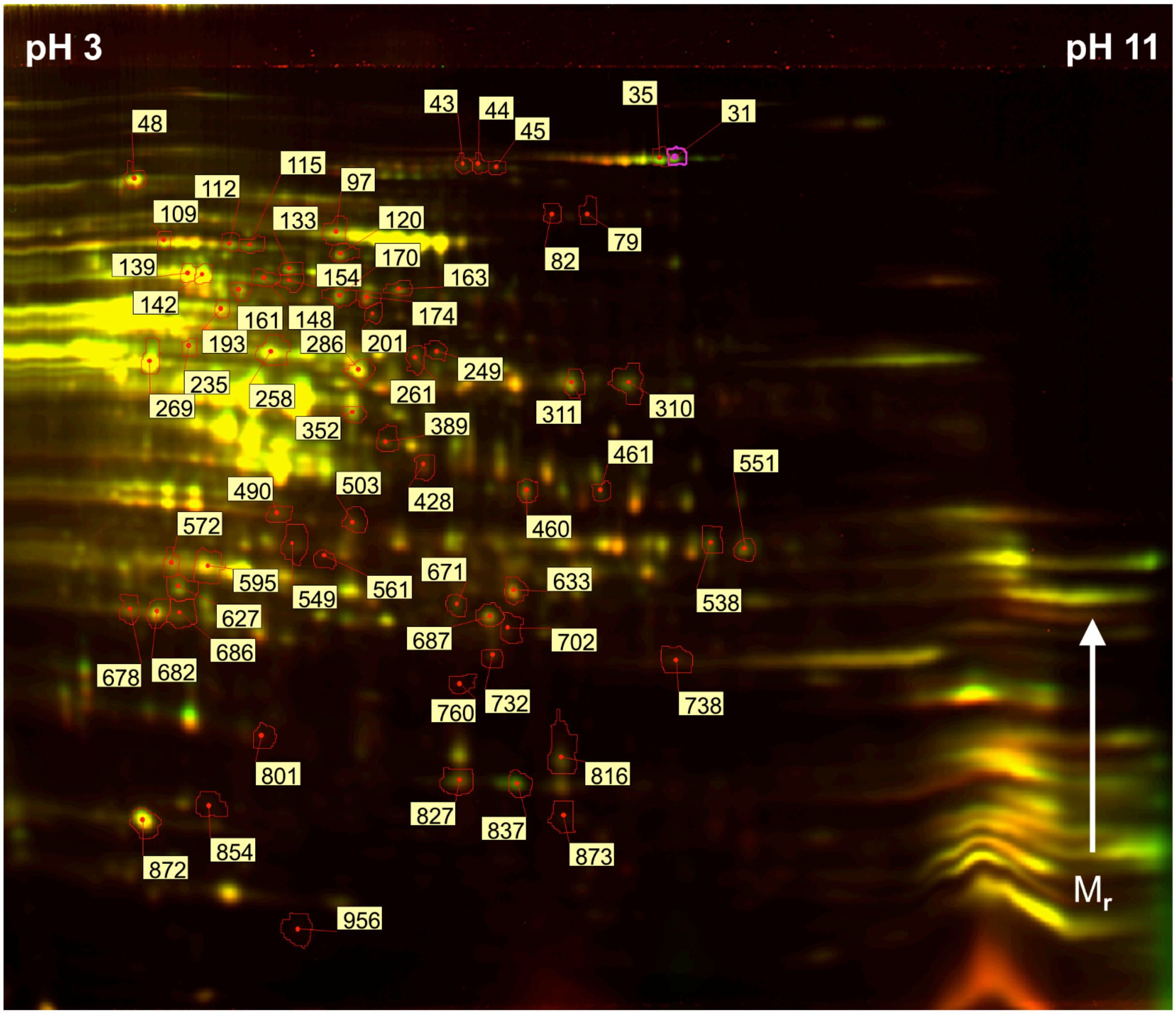

Supplementary Fig. S1

WCH16

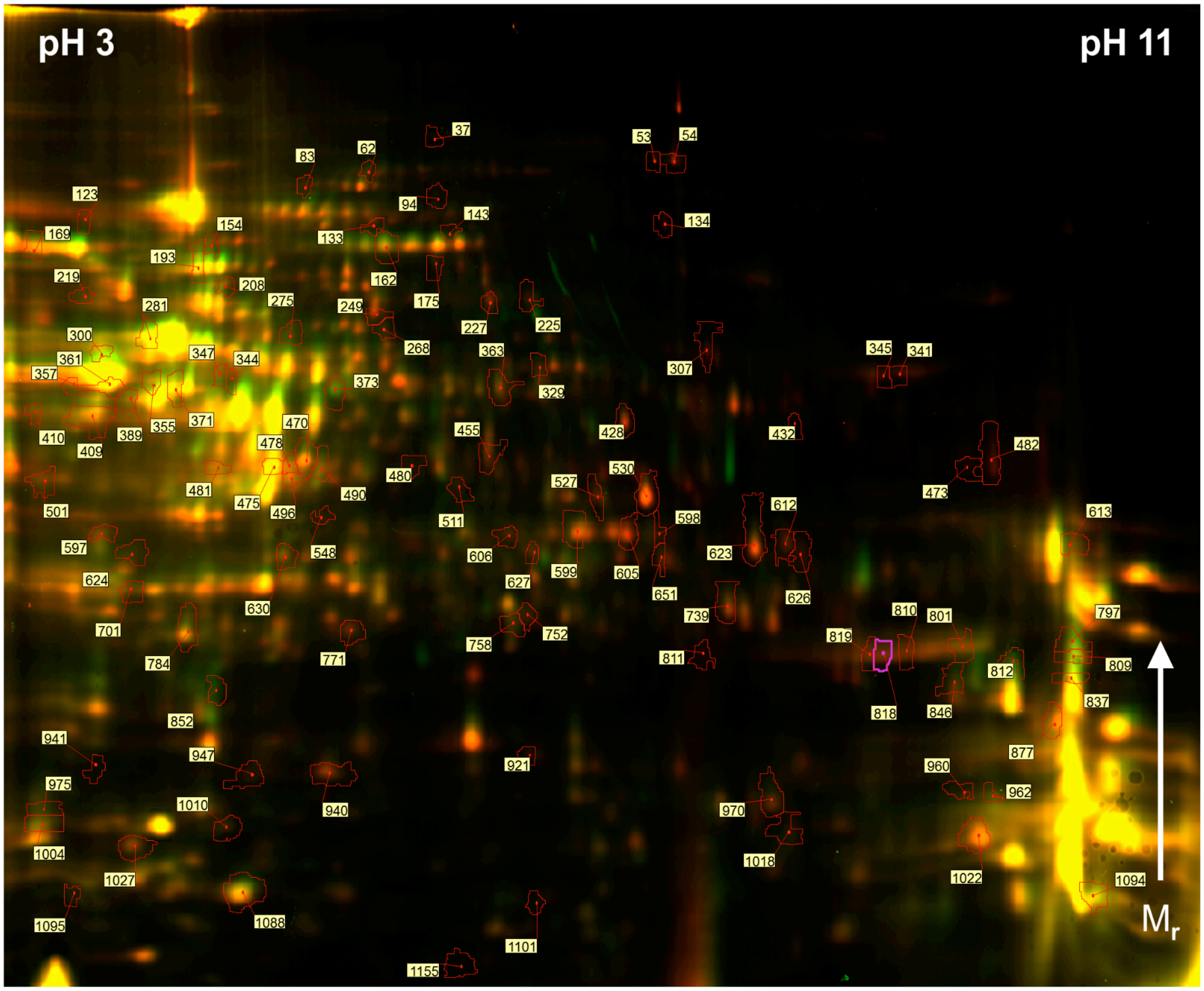

Supplementary Fig. S2

WCH43

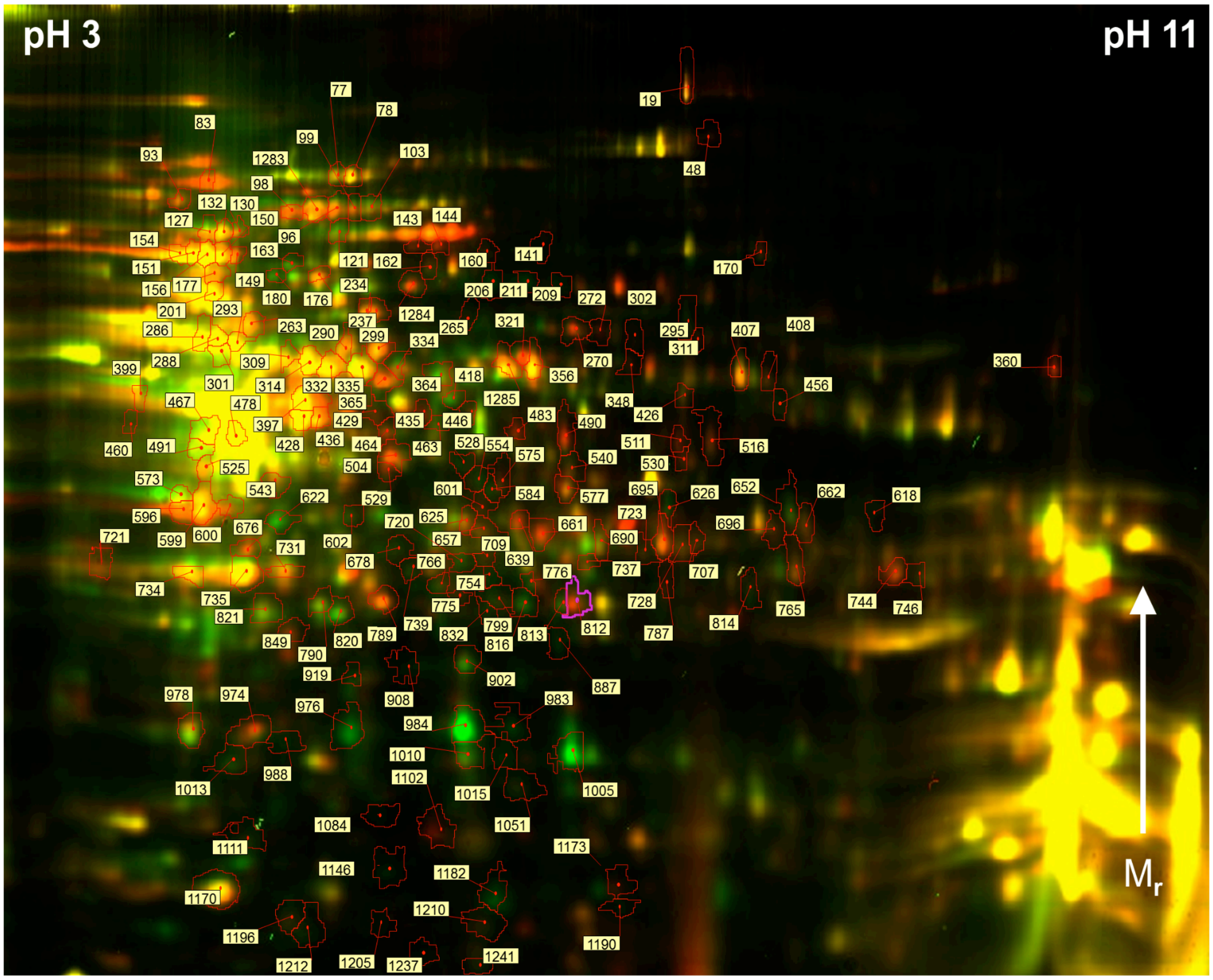

Supplementary Fig. S3
